# Distribution of Flumequine in Intestinal Contents and Colon Tissue in Pigs after Its Therapeutic Use in the Drinking Water

**DOI:** 10.3390/ani11061514

**Published:** 2021-05-23

**Authors:** Jose M. Rodríguez, M. Jose Diez, Matilde Sierra, Juan J. Garcia, Nelida Fernandez, Raquel Diez, Ana M. Sahagun

**Affiliations:** Pharmacology, Department of Biomedical Sciences, Institute of Biomedicine (IBIOMED), Veterinary Faculty, University of Leon, 24071 Leon, Spain; jmrodl@unileon.es (J.M.R.); mjdiel@unileon.es (M.J.D.); msiev@unileon.es (M.S.); jjgarv@unileon.es (J.J.G.); mnferm@unileon.es (N.F.); amsahp@unileon.es (A.M.S.)

**Keywords:** colon tissue, flumequine, intestinal content, oral administration, pig, plasma, HPLC

## Abstract

**Simple Summary:**

Gastrointestinal diseases are relatively common in pigs, and cause important economic losses. These disorders include, among others, swine dysentery, a severe diarrheal disease in growing and finisher pigs caused by *Brachyspira hyodysenteriae*. Flumequine, a fluoroquinolone indicated in the treatment of colibacillosis, enteritis and gastroenteritis, may help to minimize the impact of swine dysentery. Thus, the objective of this study was to assess flumequine concentrations in pig plasma, colon tissue and intestinal contents following oral administration through drinking water, and determine if it would be effective against the sensitive bacteria found in the colon. The drug was not detected in any plasma sample, while concentrations were higher on the days of treatment than after having finished it. Concentrations achieved in colon tissue and intestinal contents were lower than those effective against the most common intestinal pathogenic microorganisms in swine, including *B. hyodysenteriae*, suggesting that this dosage would not be effective.

**Abstract:**

Flumequine concentrations in plasma, colon tissue and intestinal contents were evaluated in 12 healthy pigs after oral administration (12 mg/kg every 24 h for 5 consecutive days in drinking water). Plasma, colon tissue and intestinal content samples were collected from animals sacrificed on days 3, 6 and 7. Concentrations were measured by high performance liquid chromatography after having validated the method, following the European Medicines Agency (EMA) requirements. The drug was not detected in any plasma sample. In colon tissue, concentrations were higher on day 3 (0.230 ± 0.033 µg/g, descending colon; 0.156 ± 0.093 µg/g, ascending colon) than on day 6 (0.187 ± 0.123 µg/g, descending colon; 0.107 ± 0.007 µg/g, ascending colon). Concentrations were considerably higher in intestinal contents, again on day 3 (1.349 ± 1.401 µg/g, descending colon; 0.591 ± 0.209 µg/g, ascending colon) than on days 6 (0.979 ± 0.346 µg/g, descending colon; 0.595 ± 0.075 µg/g, ascending colon) and 7 (0.247 ± 0.172 µg/g, descending colon; 0.172 ± 0.086 µg/g, ascending colon). Measured concentrations were lower than those effective against the most common intestinal pathogenic microorganisms in swine and, more specifically, *Brachyspira hyodysenteriae*.

## 1. Introduction

Flumequine (9-fluoruro-6,7-dihydro-5-methyl-1-oxo-1H,5H-benzo-quinolizine-2-carboxylic acid) was the first fluoroquinolone developed. This drug was transiently used in human medicine until significant adverse reactions at the ocular level were reported. In veterinary medicine, despite the availability of newer fluoroquinolones, it is commonly used in livestock to treat infections in the digestive, pulmonary and urinary tract [1,2], mainly as mass medication, due to its relatively low cost and good tolerability. This drug is especially active against a wide range of Gram-negative bacteria, including *Escherichia coli*, *Salmonella* spp. and *Pasteurella* spp. in several food-producing species [3,4,5,6,7]. Like other fluoroquinolones, this compound affects two bacterial enzymes, DNA gyrase and topoisomerase IV [8,9,10,11,12,13]. 

In pigs, gastrointestinal diseases are relatively common, and cause important economic losses, as weight gain is reduced and the feed conversion ratio worsens. In this animal species, three major regions with clinical significance are considered in the digestive system: duodenum and jejunum, for infections caused by *Escherichia coli*; ileum for those produced by *Lawsonia intracellularis*; and colon for *Brachyspira pilosicoli* and *Brachyspira hyodysenteriae* infections [14]. Among these, *B. hyodysenteriae* causes swine dysentery, a severe diarrheal disease, in growing and finisher pigs.

The possibilities to treat and control these diseases in pigs are limited. Flumequine is indicated in the treatment of colibacillosis, enteritis and gastroenteritis caused by Gram-negative bacteria [15,16,17,18,19]. As this drug is already indicated for the treatment of several intestinal diseases, it should be assessed whether flumequine would be useful as a therapeutic option against another intestinal disease such as swine dysentery, which would potentially extend its therapeutic indications. As for *B. hyodysenteriae*, although different antimicrobials are available, decreasing susceptibility has been reported worldwide [20]. The emergence of *B. hyodysenteriae* strains with reduced susceptibility to first-line treatments has led assessing new therapeutic options against this microorganism. As some authors have pointed out [21], treatment of swine dysentery is empirical, as *in vitro* susceptibility tests are not usually carried out for these microorganisms. With regards to fluoroquinolones, scarce information on the susceptibility of *Brachyspyra* spp. to these drugs. Only two studies have assessed the in vitro susceptibility of *Brachyspyra* spp. isolates to ciprofloxacin and moxifloxacin [22], and flumequine [23]. 

The pharmacokinetics of flumequine has been studied in several animal species, including pigs, cattle, sheep, goats, birds, dogs, rats and species of interest in aquaculture [4,7,24,25,26,27,28,29,30]. However, no data are available on the behavior of flumequine at the intestinal level after oral administration. This compound is administered to pigs at the oral dose of 12–15 mg/kg for 3–5 days [15,16,17,18,19].

To be clinically effective, any antibacterial agent must reach the infection site with sufficient concentrations to eliminate susceptible microorganisms. As several pathogenic microorganisms that can affect pigs are found in the colon, it is important to determine drug concentrations in this part of the large intestine, and if they would be adequate to eliminate these microorganisms. Moreover, the knowledge of colon concentrations may help to reduce the selective pressure on both endogenous and pathogenic bacteria, minimizing the spread of resistant microorganisms. In the European Union (EU), and according to the European Medicines Agency (EMA), fluoroquinolones should be reserved for the treatment of clinical conditions which have responded poorly to other classes of antimicrobials [31]. On the other hand, better dosage regimens should be established based on pharmacokinetic/pharmacodynamic (PK/PD) integration [32].

So, the aim of this study was to determine flumequine concentrations in plasma, colon tissue and intestinal contents in pigs following oral administration through drinking water at the recommended dosage, to verify if it would be effective against the sensitive bacteria found in the colon. The analytical method for determination and quantification of flumequine in pig plasma, colon tissue and intestinal contents has also been validated, according to the European Medicines Agency guideline EMA/CHMP/ EWP/192217/2009 [33].

## 2. Materials and Methods

### 2.1. Animals and Experimental Procedures

Twelve healthy male Landrace swine (6–8 weeks old) not exposed to previous antimicrobial treatment, weighing 14.7 ± 0.6 kg were used. Pigs were housed individually in 12 pens in the experimental farm of the Faculty of Veterinary (University of Leon, Leon, Spain). The relative humidity and temperature of the breeding environment were maintained at 45–65% and 18–25 °C, respectively. Swine were maintained under these conditions at least 1 week before the assay, with free access to water and chow. 

Drinking water was controlled daily before the study to establish the average daily medication. Pigs were fasted for 12 h before starting treatment.

Animals were randomized into 3 groups of 4 pigs each. All animals received the same dose (12 mg/kg every 24 h according to manufacturer’s instructions). Flumesyva^®^ 20% (Laboratories Syva, Leon, Spain) was used for oral administration. The commercial formulation was diluted in water immediately before use. The amount of drug to be administered was individually calculated and diluted to a final volume of 1 L. Pigs were dosed each morning for 5 consecutive days. Every day, the animals were weighed and the dose adjusted to increasing body weight. Medicated water was prepared fresh every 24 h. Once consumed, the animals had free access to water, with monitoring for each pig water intake daily.

### 2.2. Sample Collection

Heparinized blood samples were taken from the jugular vein immediately before sacrifice. After collection, plasma was separated by centrifugation and stored at −80 °C until analyzed.

Pigs were sacrificed at different time intervals: Animals of group 1 on day 3 (24 h after the last dose administered); group 2 on day 6 (24 h after the last dose administered) and those in group 3 on day 7 (48 h after the last dose administered). Thus, the latter two groups (2 and 3) were sacrificed 1 and 2 days after the end of treatment, respectively. Two more animals not treated with flumequine were used as controls to obtain blank samples and validate the analytical method. 

Animals were euthanized with a mix of embutramide, mebezonium iodide and tetracaine hydrochloride given intravenously (T-61^®^, Merck Sharp & Dohme Animal Health, Salamanca, Spain). The colon was excised, and its three segments identified (descending, transverse and ascending). The intestinal contents of each colon segment were carefully collected and frozen at −80 °C. The three sections in colon were washed in water, cut into 1 g pieces and frozen at −80 °C until analysis. Utmost care was taken to avoid cross-contamination between samples.

All procedures were performed in accordance with the Spanish regulations for the handling and use of laboratory animals, and conducted under the close supervision and guidance of an experienced veterinarian. The minimum number of animals and duration of observations required to obtain consistent data were employed. The Ethics Committee Report approval was obtained from the University of Leon.

### 2.3. Standards and Chemicals

Flumequine CRS was purchased from the European Pharmacopoeia Reference Standards (Council of Europe, Strasbourg, France), and oxolinic acid (internal standard, IS) from Sigma-Aldrich (Merck, Darmstadt, Germany). HPLC grade acetonitrile and ethyl acetate were obtained from Merck (Darmstadt, Germany), potassium dihydrogen phosphate was supplied from VWR International (Radnor, PA, USA) and sodium hydroxide 1M was acquired from Panreac (Barcelona, Spain). Ultrapure water was produced in our laboratory by using an Ultramatic water purification system from Wasserlab (Barbatain, Spain).

### 2.4. Determination of Flumequine

Flumequine concentrations in plasma, colon tissue and intestinal contents were determined with a method based on others previously described [34,35,36], with major modifications. All procedures were performed at room temperature.

#### 2.4.1. Plasma Samples Extraction

A total of 4 mL ethyl acetate were added to 1 mL plasma, shaken for 5 min and centrifuged at 3000 rpm for 10 min. The supernatant was then transferred to a glass tube and evaporated under a nitrogen stream. Samples were reconstituted with 1 mL mobile phase and injected into the HPLC system. 

#### 2.4.2. Colon Tissue Samples Extraction

5 mL ultrapure water were added to 1 g colon tissue samples, homogenized for 30 s (Omni-Mixer, Omni International, Kennesaw, GA, USA), and centrifuged at 3000 rpm for 10 min. The aqueous phase was collected and mixed with 4 mL ethyl acetate, shaken for 5 min and centrifuged at 3000 rpm for 10 min. Ethyl acetate was transferred into a flask. This extraction procedure was repeated twice again. The collected organic phase was almost completely evaporated in a rotary evaporator and, after that, to dryness under nitrogen stream. The residue was dissolved in 1 mL mobile phase and injected into the chromatograph system. 

#### 2.4.3. Intestinal Content Samples Extraction

5 mL ultrapure water were added to 0.5 g intestinal content, homogenized for 20 s (Omni-Mixer, Omni International, Kennesaw, GA, USA) and centrifuged at 3000 rpm for 10 min. Then, 4 mL ethyl acetate were added to the collected aqueous phase, shaken for 5 min and centrifuged at 3000 rpm for 10 min. Ethyl acetate was transferred into a flask, repeating the extraction procedure twice again. The collected organic phase was almost completely evaporated in a rotary evaporator and then to dryness under nitrogen stream. The residue was dissolved in 1 mL mobile phase and injected into the HPLC system. 

### 2.5. HPLC System and Conditions

Experimental and fortified plasma, colon tissue and intestinal content samples were analyzed for flumequine by the HPLC system Waters Alliance e2695 with photodiode array (model 2998) (Waters Corporation, Mildford, MA, USA). Chromatographic separation was performed at room temperature by using an XBridge C_18_ column (4.6 mm × 250 mm internal diameter, 5 µm, Waters). The mobile phase consisted of a mixture of potassium dihydrogen phosphate (1.36 g/L) and acetonitrile 51:49 (*v*/*v*) (pH = 4.75). Isocratic flow rate was 1 mL/min and wavelength set up at 320 nm. Injection volume was 20 µL. All procedures were performed at room temperature (column and sample carousel). In these conditions, retention times were 3.6 min (oxolinic acid, IS) and 5.1 min (flumequine). The IS was selected, taking into account the EMA guideline [33]. Oxolinic acid showed the best behavior at the detection wavelength. The study was conducted under the Good Laboratory Practice (GLP) regulations at the University of Leon (Spain).

### 2.6. Validation of the Analytical Methodology

The developed method for plasma, colon tissue and intestinal content samples was validated according to validation procedures, parameters and acceptance criteria based on the European Medicines Agency guideline EMA/CHMP/EWP/192217/2009 for the following parameters: selectivity, carry-over, lower limit of quantification (LLOQ), calibration curve, accuracy, precision, matrix effect and stability [33]. 

#### Standard Solutions, Plasma, Tissue and Intestinal Content Calibrators, and Quality Controls

Selectivity was assessed by analyzing six blank samples of plasma, tissue and intestinal contents, respectively, for interference. Carry-over was provided by injecting blank samples after the injection of a sample spiked at the highest concentration (10 µg/mL) in each run (*n* = 3). LLOQ was assessed by analyzing six samples of each type (plasma, colon tissue, intestinal content), fortified at the lowest concentration level (0.5 µg/mL). Calibration curves (*n* = 3) were established from the analysis of plasma, colon tissue or intestinal content samples spiked at six different concentration levels (0.5; 1; 2; 4; 8 and 10 µg/mL). Blank samples (biological matrix without flumequine and IS) and zero samples (biological matrix with IS) were prepared.

Quality control samples were also prepared by using blank plasma (1 mL), colon tissue (1 g) or intestinal contents (0.5 g) at concentrations of 0.5, 1.5, 5 and 7.5 µg/mL for flumequine and 10 µg/mL for IS.

Quantification was achieved by plotting the ratio of flumequine to IS as a function of flumequine concentration. The equation was achieved by using a least-squares linear regression. The back calculated concentration of the calibration standard was checked to be in agreement with EMA guidance criteria (accuracy within ±15% of a nominal value), except for LLOQ (±20%). At least 75% of the calibration standards, with a minimum of six calibration standard levels, must fulfil this criterion [33]. 

The linearity of the standard curve (0.5–10 µg/mL) was checked by calculating the coefficient of determination (R^2^). Within-run and between-run precision and accuracy of the current methods were evaluated by analyzing five samples of each biological matrix at four concentration levels (0.5, 1.5, 5 and 7.5 µg/mL). Precision was evaluated by calculating the coefficient of variation (CV), and accuracy reported as a nominal concentration of analyte and expressed as a percentage. 

The stability of the stock was assessed and working solutions of flumequine and IS and spiked samples was performed at two concentration levels (1.5 and 7.5 µg/mL) under different storage conditions: at room temperature for 24 h; at 4–8 °C for 24 and 48 h; and at −80 °C for 24, 48 and 72 h, and 5 days. Freeze and thaw stability of flumequine were determined in matrix and spiked samples frozen at days 2, 3 and 5 at −80 °C (1, 2 and 4 cycles). All experiments were conducted in 2 replicates for each concentration level. All samples were fully thawed at room temperature. 

## 3. Results

According to the European guidelines [33], the essential characteristics of a bioanalytical method to ensure the acceptability of the performance and reliability of the analytical results are selectivity, lower limit of quantification (LLOQ), response function and calibration range, accuracy, precision, matrix effects, stability of the analyte in the biological matrix and stability of the analyte and the IS in the stock and working solutions, as well as in extracts during the entire storage period and processing conditions.

The method validated for the quantification of flumequine in pig plasma, colon tissue and intestinal contents were selective, precise, accurate, repeatable and robust. The selectivity of the method was demonstrated. No significant interferences were detected for any of the sample types at the retention time of flumequine (see supplementary file, Figures S1, S2 and S3). No carry-over occurred after the analysis of samples with flumequine at high concentrations. The peak height of flumequine and IS in blank samples injected after high concentration samples was in accordance with the EMA requirements (higher than 20% of the LLOQ and 5% for the IS). 

The LLOQ of flumequine was 0.5 µg/mL in all biological matrices, and the limit of detection (LOD) was established in 0.25 µg/mL for plasma and 0.1 µg/mL for colon tissue and intestinal contents. The characteristics of the calibration curves are summarized in Table 1 (see also supplementary file, Figures S4, S5 and S6). Analytical procedures were lineal, ranging drug recovery from 70.2% in intestinal contents to 94.8% in plasma.

For plasma samples (Table 2), within-run and between-run accuracy were in the range of 92.9–108.0% and 93.2–104.5%, respectively. Within-run variation coefficient (CV) was 4.5–11.9% and between-run CV 6.4–8.2%. In colon tissue, within-run and between-run accuracy were in the range of 93.8–105.6% and 94.3–104.6%, respectively, whereas within-run CV was 2.2–6.2% and between-run CV was 2.6–6.6%. Finally, in intestinal contents, within-run and between-run accuracy were in the range of 95.1–109.3% and 96.9–105.1%, respectively, and within-run CV was 0.9–5.4% and between-run CV was 2.6–5.7%. Thus, good accuracy and precision of the analytical methods used were proved.

The stock solution of flumequine and IS was stable at room temperature for 24 h, at 4–8 °C for 48 h, and at −80 °C for 5 days, as well as the working standard solutions, being less than 10% as the difference between the peak height of fresh prepared and that of the stored solutions. Flumequine was stable in pig plasma, colon tissue and intestinal contents for all temperatures and times analyzed, with CV always <15% of the nominal concentration.

### Determination of Flumequine in Plasma, Colon Tissue, and Intestinal Contents

The validated method was successfully applied to determine and quantify flumequine in pig plasma, colon tissue, and intestinal contents after treatment with 12 mg/kg every 24 h for 5 consecutive days. Drug concentrations were determined in each colon segment of all animals (descending, transverse and ascending).

With regard to plasma samples, no detectable concentrations of flumequine were measured in any sample. As for colon tissue and intestinal content samples, concentrations are summarized in Table 3 for each colon segment. 

In colon tissue samples, flumequine was mostly quantified in samples from days 3 and 6 (the drug was not detected in some colon segments of one animal sacrificed on day 3). The highest concentrations were seen in samples obtained on day 3, and were mostly reached in descending colon tissue. Nevertheless, flumequine concentrations were always outside the validated concentration range. On day 7, flumequine had already been completely eliminated, and it was detected in no colon segment. Finally, in intestinal content samples, concentrations were considerably higher than those quantified in the colon tissue. The drug was measured mostly on animals sacrificed on days 3 and 6. Moreover, the highest drug concentrations were also found in those animals sacrificed during treatment (day 3), and in those samples taken from the descending or transverse segments, although some values were below the validated range. In animals sacrificed on day 7, flumequine was detected in lower concentrations, mostly outside the validated range of the analytical method used.

## 4. Discussion

Fluoroquinolones are very potent antimicrobials widely used in veterinary practice for the treatment of several intestinal and respiratory infections in livestock, including pigs [37]. To the best of the authors’ knowledge, this is the first time that flumequine concentrations have been determined in colon tissue and intestinal contents in this animal species. To determine drug concentrations, we developed and validated a method which is sensitive and reliable, and complies with the EMA guidelines for bioanalytical method validation [33]. Although the pharmacokinetics of flumequine has been studied in several animal species, data in pigs are scarce. Only Villa et al. [7] described the intravenous and intramuscular disposition of this drug in swine, and no data are available on its pharmacokinetic behavior after oral administration. As these authors pointed out, among other factors, the scarcity of pharmacokinetic data for antibacterial drugs in swine could be related to the difficulties inherent in inserting an intravenous catheter to obtain blood samples. Thus, the analytical validated method may be used to determine flumequine concentrations in pig plasma and tissues.

In the study reported here, the colon disposition of flumequine after oral administration at the recommended dosage (12 mg/kg every 24 h for 5 consecutive days as drinking water) was established. In plasma samples, drug concentrations were not detected on days 3, 6 and 7. Regarding intestinal tissue samples, peak concentrations were achieved on day 3, decreasing in the following days until undetected on day 7. Concentrations were also higher in the more proximal sections of the colon. Nevertheless, measured drug concentrations were always below the LLOQ. Thus, they should be interpreted with caution. As for intestinal content concentrations, they greatly exceeded plasma and colon tissue concentrations, as described for other quinolones in the same animal species [38], sheep [39] and chickens [40]. These differences in concentrations could result from different drug binding capacities of tissues and intestinal contents [41]. High percentages of enrofloxacin in pigs (42%) [41] and norfloxacin in human beings (95%) [42] were inactivated because of this binding. On the other hand, the first-pass effect may also contribute to the lower concentrations found in colon tissue. In pigeons, Dorrestein et al. [25] pointed to the possibility of the first-pass effect as well as its inactivation in the digestive tract preceding absorption as contributing factors to explain the low bioavailability of flumequine after oral administration. However, this first-pass effect has not been observed in cattle [27]. Intestinal transporters may also play an important role in drug secretion from the blood back into the intestine [32]. Such a mechanism has been described for ciprofloxacin, norfloxacin and pefloxacin [43,44], being involved different transporters depending on the fluoroquinolone assessed (sparfloxacin is a P-glycoprotein substrate, whereas for ciprofloxacin the process is mediated by organic anion and/or cation transporters) [43]. Again, none of these mechanisms has been described for flumequine. 

On the other hand, a high variability in measured concentrations were observed, which is common when antimicrobials are administered to pigs as water medication [45]. Mass medication of veterinary drugs through drinking water is frequently used in this animal species. Although our study was carried out under very controlled conditions, it is critical how quickly the drug is ingested, and would explain if concentrations rise more or less rapidly and, consequently, concentrations attained are higher or lower [45].

The use of fluoroquinolones in livestock mass medication is a matter of concern [46]. It is recognized that group administration of antimicrobials plays an important role in the selection of resistant bacteria [47]. Although limited information is available on intestinal exposure for the antimicrobials currently marketed, it is known that it could influence the selection of resistant bacteria [48]. In fact, incomplete oral absorption may affect the intestinal microbiota [48]. Several studies have highlighted that the increasing level of antimicrobial resistance in gut microbiota was mainly due to the antimicrobial misuse [49,50]. Thus, optimizing the recommended dose regimen is a suitable strategy to reduce antimicrobial resistance development without jeopardizing therapy efficacy. According to the minimum inhibitory concentration (MIC) reported by Aller–Moran et al. [23] for *B*. *hyodysenteriae* (MIC_50_ = 50 µg/mL), the dosage regimen recommended for flumequine would not be sufficient to kill this pathogenic microorganism. However, the low concentrations detected could act on other intestinal resident bacteria that can influence the pathogenicity of *B. hyodysenteriae* (*Prevotella* spp. or *Bacteroides* spp.) [51]. For other intestinal pathogenic microorganisms such as *Salmonella typhimurium* or *Escherichia coli*, MIC values were also higher than the flumequine concentrations quantified (MIC_50_ = 2 and 32 µg/mL, respectively) [7] in almost all samples. Thus, and based on the results obtained, the dosage of 12 mg/kg every 24 h for 5 consecutive days would be ineffective to achieve adequate flumequine intestinal concentrations against the main colonic pathogenic microorganisms, especially *B. hyodysenteriae*. Further studies should be conducted to assess the pharmacokinetics behavior of this drug in pigs, and to reevaluate the dosage scheme used.

## 5. Conclusions

The study reports the development and validation of a reliable, selective and robust HPLC method for the quantification of flumequine in swine plasma, colon tissue and intestinal contents according to EMA guidelines. This is the first study that determines flumequine concentrations in swine plasma, colon tissue and intestinal contents after oral administration. At the recommended dosage (12 mg/kg every 24 h for 5 days, drinking water), the drug was not detected in pig plasma, and low concentrations have been quantified in colon tissue and intestinal contents. The results of our study suggest this dosage would not be effective in pigs against the main pathogenic microorganisms present in the colon. Rational drug use is essential to prevent the emergence of antimicrobial resistance and to improve the clinical response. Further studies, including the oral pharmacokinetics, are necessary to fully evaluate its therapeutic possibilities in this animal species.

## Figures and Tables

**Table 1:** Data from linear regression analysis of calibration curves.

Characteristic	Plasma	Colon Tissue	Intestinal Content
Curve 1 (R^2^)	y = 0.098 x + 0.007 (0.996)	y = 0.098 x + 0.008 (0.998)	y = 0.064 x + 0.005 (0.998)
Curve 2 (R^2^)	y = 0.088 x + 0.005 (0.992)	y = 0.098 x + 0.003 (0.999)	y = 0.064 x + 0.013 (0.998)
Curve 3 (R^2^)	y = 0.087 x + 0.004 (0.990)	y = 0.087 x + 0.021 (0.987)	y = 0.065 x + 0.016 (0.998)
LLOQ (µg/mL)	0.5	0.5	0.5
LOD (µg/mL)	0.25	0.1	0.1
Recovery (%) (Mean ± SD)	94.8 ± 4.7	91.8 ± 9.2	70.2 ± 4.2

SD: standard deviation.

**Table 2:** Within-run and between-run accuracy and precision for the samples processed ^a^.

Matrix	Nominal Concentration (µg/mL)	Accuracy (% from Nominal Concentration)		Precision (% CV)	
		Within-Run (Range)	Between-Run	Within-Run (Range)	Between-Run
Plasma	0.5	98.6–108.0	104.5	4.5–11.9	8.2
	1.5	95.8–101.1	99.3	4.9–6.8	6.4
	5	92.9–93.5	93.2	6.0–10.0	7.2
	7.5	95.4–95.9	95.6	5.1–9.5	6.5
Colon tissue	0.5	103.4–105.6	104.6	5.4–6.2	5.2
	1.5	97.9–107.4	101.3	3.8–5.9	6.6
	5	93.8–94.9	94.3	2.8–4.2	3.6
	7.5	97.1–98.4	97.9	2.2–3.0	2.6
Intestinal content	0.5	100.0–109.3	105.1	4.2–5.2	5.7
	1.5	99.2–104.7	101.7	0.9–5.4	3.8
	5	97.7–102.1	99.5	2.8–4.0	3.7
	7.5	95.1–99.2	96.9	1.3–1.8	2.6

^a^ 15 replicates.

**Table 3:** Flumequine concentrations in colon tissue and intestinal contents in pigs after oral administration with drinking water (individual concentrations, and mean ± SD).

Animal		Flumequine Concentrations (µg/g)					
		Colon Tissue			Intestinal Content		
		Descending	Transverse	Ascending	Descending	Transverse	Ascending
3 days	1	0.261 ^a^	0.306 ^a^	0.264 ^a^	3.392	1.653	0.442 ^a^
	2	0.231 ^a^	-	-	0.227 ^a^	0.200 ^a^	-
	3	0.244 ^a^	0.192 ^a^	0.103 ^a^	1.022	1.732	0.501
	4	0.185 ^a^	0.173 ^a^	0.102 ^a^	0.756	1.085	0.829
	Mean ± SD	0.230 ± 0.033	0.224 ± 0.072	0.156 ± 0.093	1.349 ± 1.401	1.168 ± 0.706	0.591 ± 0.209
6 days	5	0.143 ^a^	0.136 ^a^	0.104 ^a^	1.436	1.167	0.503
	6	0.131 ^a^	0.129 ^a^	0.105 ^a^	1.030	1.021	0.638
	7	0.103 ^a^	0.104 ^a^	0.117 ^a^	0.627	0.530	0.567
	8	0.370 ^a^	0.127 ^a^	0.101 ^a^	0.822	0.910	0.671
	Mean ± SD	0.187 ± 0.123	0.124 ± 0.014	0.107 ± 0.007	0.979 ± 0.346	0.907 ± 0.272	0.595 ± 0.075
7 days	9	-	-	-	0.437 ^a^	0.653	0.111 ^a^
	10	-	-	-	0.100 ^a^	0.303 ^a^	-
	11	-	-	-	-	0.120 ^a^	-
	12	-	-	-	0.205 ^a^	0.253 ^a^	0.232 ^a^
	Mean ± SD	-	-	-	0.247 ± 0.172	0.332 ± 0.227	0.172 ± 0.086

^a^ Concentrations under LLOQ; - concentrations under LOD; SD: standard deviation.

## Data Availability

Data are available on request the corresponding author.

## Supplementary Materials

The following are available online at https://www.mdpi.com/article/10.3390/ani11061514/s1, Figure S1: Representative HPLC chromatogram of (a) blank plasma sample; (b) plasma sample fortified with flumequine (10 μg/mL) and IS (10 μg/mL)., Figure S2: Representative HPLC chromatogram of (a) blank colon tissue sample; (b) colon tissue sample fortified with flumequine (10 μg/mL) and IS (10 μg/mL), Figure S3: Representative HPLC chromatogram of (a) blank intestinal content sample; (b) intestinal content sample fortified with flumequine (10 μg/mL) and IS (10 μg/mL), Figure S4: Plasma calibration curve (Run 1), Figure S5: Colon tissue calibration curve (Run 1), Figure S6: Intestinal content calibration curve (Run 1).
