# Supplementary material for: Distribution of Flumequine in Intestinal Contents and Colon Tissue in Pigs after Its Therapeutic Use in the Drinking Water
Source: Animals (Basel). 2021 May 23;11(6):1514. doi: 10.3390/ani11061514 (PMC8224771; doi:10.3390/ani11061514)
Supplement: Supplementary file 1 [file animals-11-01514-s001.zip › animals-1205599-SI.pdf]

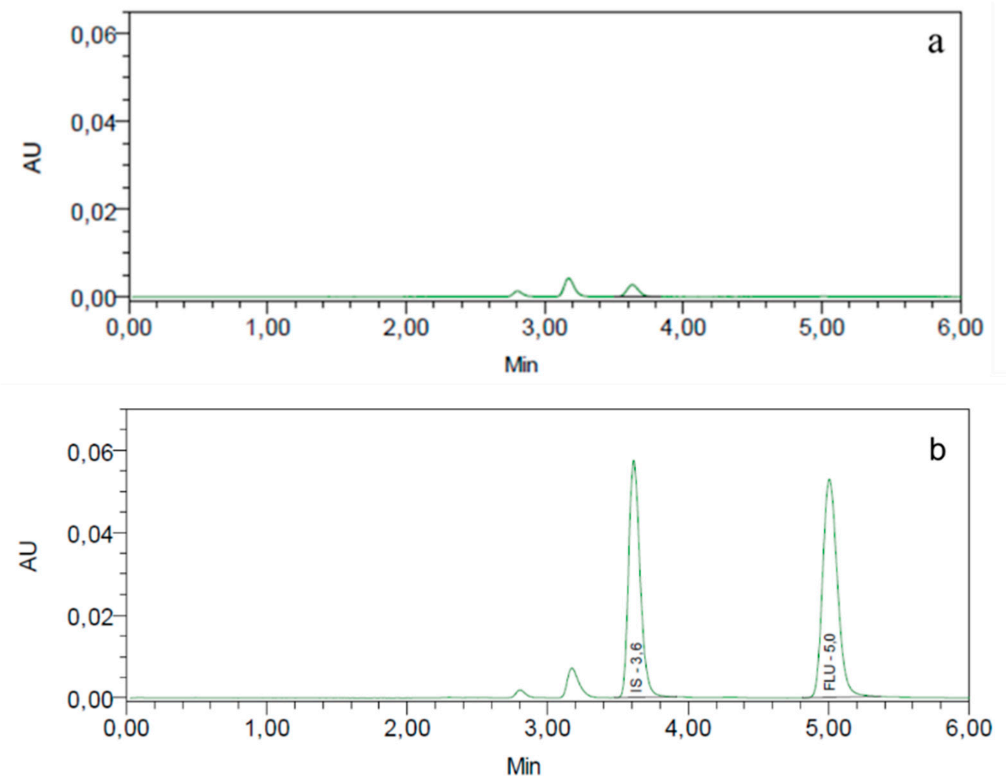

Supplementary Figure S1. Representative HPLC chromatogram of (a) blank plasma sample; (b) plasma sample fortified with flumequine (10 μg/mL) and IS (10 μg/mL).

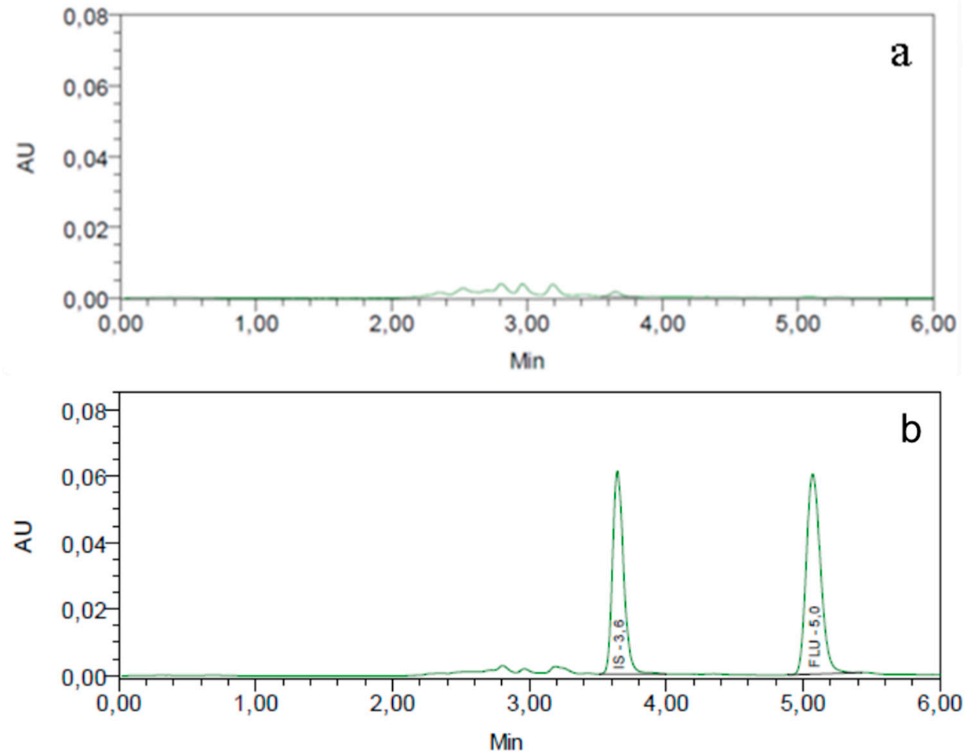

Supplementary Figure S2. Representative HPLC chromatogram of (a) blank colon tissue sample; (b) colon tissue sample fortified with flumequine (10 μg/mL) and IS (10 μg/mL).

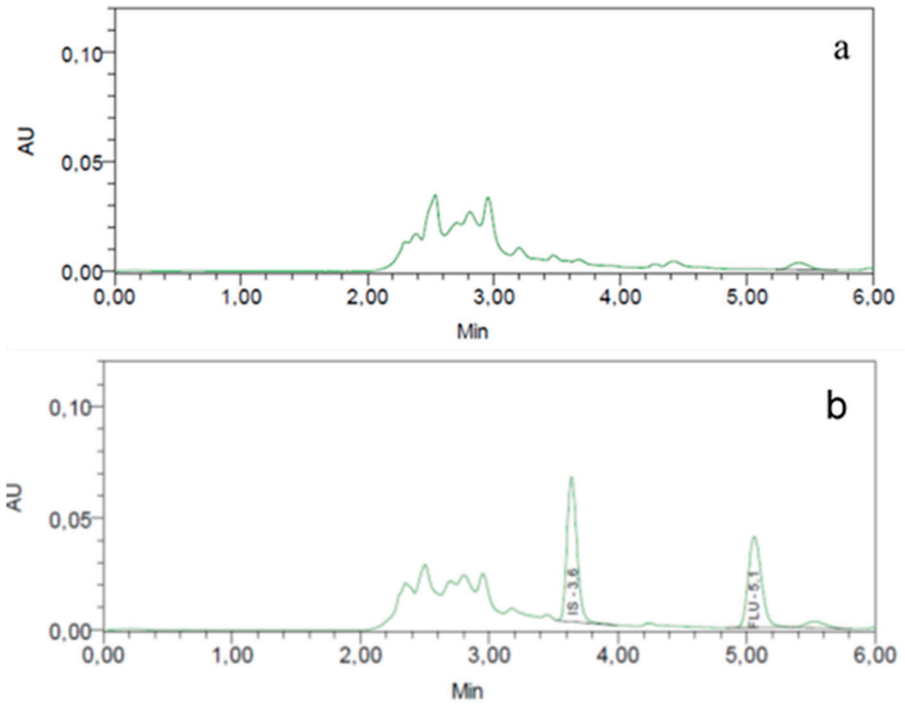

Supplementary Figure S3. Representative HPLC chromatogram of (a) blank intestinal content sample; (b) intestinal content sample fortified with flumequine (10 μg/mL) and IS (10 μg/mL).

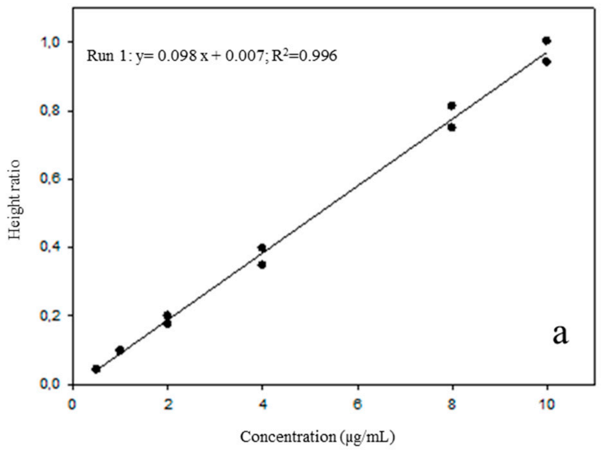

Supplementary Figure S4. Plasma calibration curve (Run 1).

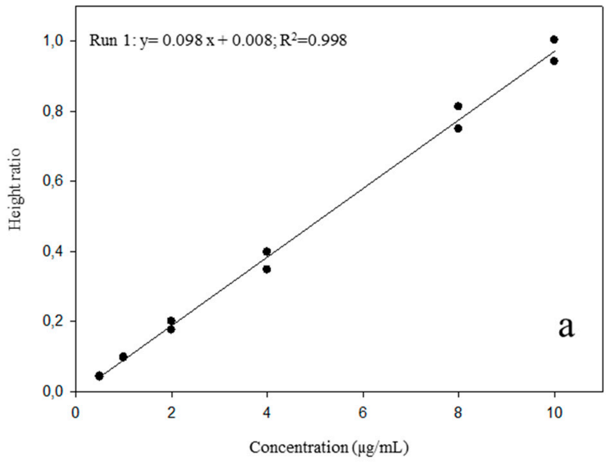

Supplementary Figure S5. Colon tissue calibration curve (Run 1).

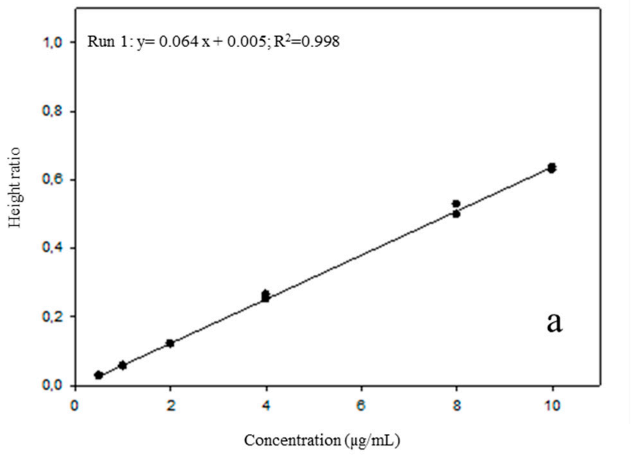

Supplementary Figure S6. Intestinal content calibration curve (Run 1).
